# Impact of the COVID-19 Pandemic on Contraceptive Services at Selected Primary Health Care Facilities in India, Nigeria, and Tanzania: Cross-Sectional Study

**DOI:** 10.2196/59874

**Published:** 2025-12-02

**Authors:** Rita Kabra, Beena Joshi, Ester Elisaria, Tanimola Makanjuola Akande, Komal Preet Allagh, Adesola Olumide, Deepti Tandon, Ranjan Prusty, Mary Ramesh, Donat Shamba, Bhavya MK, Shabana Khan, James Kiarie

**Affiliations:** 1 Department of Sexual and Reproductive Health and Research, including UNDP/UNFPA/UNICEF/WHO/World Bank Special Programme of Research, Development and Research Training in Human Reproduction, World Health Organization, Avenue Appia 20, Geneva, 1211, Switzerland, 41 796036224; 2 Department of Operational and Implementation Research, ICMR National Institute for Research in Reproductive and Child Health, Mumbai, India; 3 Department of Health System Impact Evaluation and Policy, Ifakara Health Institute, Dar es Salaam, United Republic of Tanzania; 4 Department of Epidemiology and Community Health, University of Ilorin, Ilorin, Nigeria; 5 Institute of Child Health, University of Ibadan, Ibadan, Nigeria; 6 Department of Clinical Research, ICMR-National Institute for Research in Reproductive and Child Health, Mumbai, India; 7 Department of Biostatistics, ICMR- National Institute for Research in Reproductive and Child Health, Mumbai, India

**Keywords:** facility assessment, COVID-19 pandemic, family planning, contraceptives, India, Nigeria, Tanzania, World Health Organization, contraceptive, COVID-19, primary health care, mixed method

## Abstract

**Background:**

The COVID-19 pandemic disrupted sexual and reproductive health services, including family planning (FP) and contraceptive services. The World Health Organization conducted a multicountry study in India, Nigeria, and Tanzania to determine the impact of the pandemic on the health system’s readiness to provide contraception services and trends in contraceptive uptake.

**Objective:**

This study aimed to determine the status, availability, and health facility readiness to provide contraceptive services and to compare trends in contraceptive uptake before and during the pandemic.

**Methods:**

This cross-sectional study was conducted by the Indian Council of Medical Research-National Institute of Research in Reproductive and Child Health (India), the University of Ilorin Teaching Hospital (Nigeria), and the Ifakara Health Institute (Tanzania). A total of 50 primary health facilities (11 in India, 6 in Nigeria, and 33 in Tanzania) were evaluated using a standardized facility assessment questionnaire, completed by the most knowledgeable senior health care provider or administrator at the facility. Monthly data on service utilization and contraceptive availability were collected to capture trends before and during the COVID-19 pandemic. Data were collected from May to August 2022. The study received ethical and scientific approval from the World Health Organization Ethics Review Committee and Research Project Review Panel and national regulatory bodies. Key outcomes included availability of FP guidelines and tools, service disruptions including contraceptive and abortion services, stock-outs, reasons for service disruptions, and mitigation measures to sustain service deliveries. Descriptive analysis was used to summarize the key trends and patterns.

**Results:**

Health facilities in all three countries reported shortages of various contraceptives. Contraceptive services were partially disrupted in 91% facilities in India, 83% facilities in Nigeria, and 43% facilities in Tanzania. Abortion services were partially disrupted in all surveyed facilities offering these services in India and Nigeria and in 26.7% of facilities in Tanzania. Client visits declined in health facilities in 2020 compared to 2019 in India (30%) and Nigeria (11%), with a gradual recovery thereafter. In contrast, Tanzania experienced a 1% decline in client visits in 2020. Readiness measures such as telemedicine, task shifting, community outreach, triaging, and patient redirection were implemented to minimize service disruptions.

**Conclusions:**

This study provides crucial insights into the challenges posed by the COVID-19 pandemic on contraceptive services and the measures taken to alleviate them. The findings can help countries to better prepare to prevent the disruption of FP and contraceptive services in future pandemics or emergencies.

## Introduction

The COVID-19 outbreak was first reported in December 2019, rapidly spreading across the globe and being declared a pandemic by the World Health Organization (WHO) on March 11, 2020 [1]. In an effort to curb the virus’s transmission, countries implemented various measures such as international travel restrictions, social distancing, and lockdowns. The virus’s highly dynamic nature posed challenges for taking standard coping measures in response to the pandemic. Every nation responded differently to the pandemic. Countries worldwide mobilized their resources to diagnose and treat COVID-19 patients while implementing public health strategies to contain the spread of the virus. Although necessary, this response could disrupt the provision of sexual and reproductive health (SRH) services, including family planning (FP) and contraceptive services, especially in low- and middle-income countries, where health systems are more vulnerable.

Several studies show that the COVID-19 pandemic had a major impact on the availability and provision of contraception, abortion, postabortion, and other sexual health services [2-4]. Supply chain disruptions limited the production, distribution, and availability of contraceptive commodities, resulting in stockouts [5]. Some health care facilities reduced or stopped offering their services [6], some health care providers were redirected to COVID-19 duties [7], and many women were unable to visit health care facilities due to lockdowns or fear of exposure to COVID-19 [8]. Travel to hospitals was disrupted by movement restrictions or reductions in health workers as a result of increasing numbers becoming infected with COVID-19 [9]. This created significant access and availability issues for many individuals who required these services. If the contraceptive needs of women and couples are unmet, unwanted pregnancies will increase, with long-term consequences for both women and their families [10]. It was estimated that significant levels of lockdown-related disruption over 6 months would leave 47 million women in low- and middle-income countries unable to use modern contraceptives, leading to a projected 7 million additional unintended pregnancies [11]. Furthermore, studies have modeled that even a 10% reduction in essential SRH services could lead to an estimated 15 million unintended pregnancies, 3.3 million unsafe abortions, and 29,000 additional maternal deaths in 12 months [10,12,13].

Our study aimed to determine the impact of the COVID-19 pandemic on the health system’s capacity to provide contraception services in India, Nigeria, and Tanzania. These countries were selected because they were part of the World Health Organization’s FP Accelerator Project [14], with expressed interest from the Ministries of Health to conduct the study. In this paper, we present findings of a health facility assessment, examining the availability of contraceptive services at primary health facilities and their readiness to provide these services during the COVID-19 pandemic. Additionally, we analyze and compare trends in contraceptive uptake in the 3 countries, both before and during the pandemic.

## Methods

### Study Objectives

The larger study used a mixed methods approach, using both quantitative and qualitative methods. The detailed methods and objectives of the overall study can be found in the published study protocol [15]. There were no deviations from the published protocol. In this paper, we present findings from the cross-sectional health facility assessment (quantitative study). This assessment was conducted in primary health facilities across India, Nigeria, and Tanzania. The primary objectives of the health facility assessment were to (1) determine the status, availability, and health facility readiness to provide contraceptive services in regions or localities most affected during the COVID-19 pandemic and (2) assess trends in the provision and uptake of contraceptive services in the facilities over the past 12 months. In this paper, we compare the findings of the health facility assessment from the 3 countries.

### Study Design

This study was a cross-sectional health facility assessment using a structured questionnaire. Health care providers from each health facility were purposively selected to assist in completing the questionnaire. As part of the health facility assessment, we reviewed records related to contraception services to determine the availability, type, and range of contraceptive methods offered.

### Settings

In India, this study was conducted by the Indian Council of Medical Research-National Institute of Research in Reproductive and Child Health, in Nigeria by the University of Ilorin Teaching Hospital, and in Tanzania by the Ifakara Health Institute. In the selected geographies, representative rural and urban primary health care (PHC) facilities with qualified staff providing contraception services were selected.

In India, Thane district in the State of Maharashtra was selected because it was one of the worst affected districts by the COVID-19 pandemic in Maharashtra [1]. Administratively, Thane district is divided into 7 talukas. A large part of this district is urban and is administratively governed by 6 municipal corporations. One taluka from an urban area (Navi Mumbai Municipal Corporation) and one from the rural area (Ambernath) were randomly selected [16]. In each taluka, all public health facilities were considered for the study.

In Nigeria, 3 zones (ie, South-West, North-Central, and North-West) of the 6 geopolitical zones were selected purposively. Within each zone, 1 state that is a site for Gates-funded SRH interventions (the Nigerian Urban Reproductive Health Initiative intervention and the Challenge Initiative) was selected for the study: Kwara (North-Central), Oyo (South-West), and Kano (North West) states. Two high-volume PHC facilities that were providing maternal and child health and FP services were selected within each state (1 in an urban and 1 in a rural/periurban LGAs).

In Tanzania, 3 districts from 3 regions were selected: Temeke district (Dar es Salaam), Nyamagana district (Mwanza), and Moshi urban district (Kilimanjaro). Moshi urban and Temeke districts were selected as they were a part of the Service Availability and Readiness Assessment and belonged to regions with a high number of COVID-19 cases. The Nyamagana district was chosen as it was thought that COVID-19 would impact the urban population more because of their frequent social interactions. All government hospitals, health centers, and a random sample of dispensaries that offered FP services were selected in each district. Data collection was done between May 2022 and August 2022.

### Participants

In each health facility, the most knowledgeable respondent about FP and contraceptive services was identified in consultation with the facility-in-charge. This individual was typically a senior health care provider or facility administrator. To be eligible, respondents had to meet the following inclusion criteria: (1) employed at the facility for at least 6 months before the onset of the COVID-19 pandemic, (2) directly involved in the provision of FP and contraceptive services, and (3) knowledgeable about the facility’s service readiness and availability of FP and contraceptive services.

### Assessments

A structured questionnaire was developed to assess the readiness of health facilities to provide FP and contraceptive services during the pandemic. The questionnaire was adapted from the WHO’s SARA tool [17] and the health facility checklist developed by the WHO Department of Health Emergency [18]. Key domains assessed included policies and plans, service maintenance and referrals, facility infrastructure, human resources, and availability of commodities. The questionnaire was translated into local languages at each study site: Hindi and Marathi in India, Yoruba and Hausa in Nigeria, and Kiswahili in Tanzania.

The primary outcomes of the facility assessment included the proportion of health facilities with FP guidelines and tools available, proportion of facilities where regular health services were disrupted during the pandemic, proportion of facilities that maintained FP services as part of essential health services, stockouts of contraceptives during the pandemic, reported reasons for disruption in FP service delivery (eg, interruption of contraceptive services due to facility closures or staff reassignments and supply chain disruptions affecting the availability of contraceptives), and measures taken by countries to mitigate the impact of the pandemic on FP service provision.

### Study Size

Health facilities in each country were purposively selected based on geographical location (eg, ease of access and areas with a high incidence of COVID-19 cases), organization of FP and contraceptive service delivery, and epidemic status, where the COVID-19 pandemic was likely to have significantly disrupted service delivery. A total of 50 health facilities were assessed across the 3 countries: 11 in India, 6 in Nigeria, and 33 in Tanzania. Of the 33 health facilities in Tanzania, 3 were government hospitals, 9 were health centers, and 21 were dispensaries. In Nigeria, 4 PHC facilities, 1 district health unit, and 1 maternal and child health facility were included. Of the 11 facilities in India, 5 were urban health posts, 4 were PHCs, 1 was district hospital, and 1 was rural hospital. Table 1 presents the details of the study sites in each country.

**Table 1.** Description of study sites in India, Nigeria, and Tanzania.

Country and location	Setting
India (n=11)	
Navi Mumbai Municipal Corporation area, Thane district (urban)	5 urban health posts (PHCs^a^ in urban areas) and 1 district hospital
Ambernath block (rural)	4 PHCs and 1 rural hospital
Nigeria (n=6)	
Kwara state, Ilorin LGA^b^	1 PHC (urban)
Kwara state, Afon LGA	1 district health unit (rural)
Oyo state, Ibadan north	1 PHC (urban)
Oyo state, Akinyele	1 PHC (periurban)
Kano state, Dala LGA	1 MCH^c^ (urban)
Kano state, Kura LGA	1 PHC (rural)
Tanzania (n=33)	
Mwanza, Nyamagana district	6 dispensaries, 2 health centers, and 1 hospital
Kilimanjaro, Moshi urban district	7 dispensaries, 2 health centers, and 1 hospital
Dar es Salaam, Temeke district	8 dispensaries, 5 health centers, and 1 hospital

a PHC: primary health care.

b LGA: local government areas.

c MCH: maternal and child health.

### Data Analysis

In India, data from facility assessments were entered into an electronic database and analyzed using SPSS (Statistical Package for Social Sciences). In Nigeria, the KoboCollect app was used on the research staff’s phones to collect data, and it was analyzed using SPSS version 21, whereas in Tanzania, data were collected digitally on tablets using the Open Data Kit platform and analyzed using STATA version 15 software. Descriptive analysis was used to compare the characteristics of the various facilities, including the number of clients, the type of services provided, and the contraceptive stocks.

### Ethical Considerations

The WHO Research Project Review Panel granted scientific approval, whereas the WHO Ethics Review Committee granted ethical approval (protocol IDs CERC.0103K, CERC.0103J, and CERC.0103I). In India, the Institutional Ethical Committee of Indian Council of Medical Research-National Institute of Research in Reproductive and Child Health, Mumbai (D/ICEC/Sci-166/175/2021) approved the study protocol. Additionally, permission was obtained from state and district government health officials to conduct the study at the selected sites. In Nigeria, the study was approved by the National Health Research Ethics Committee of Nigeria (NHREC/01/01/2007-07/09/2021), the University of Ilorin Ethical Research Committee (in Kwara state), and State Ministries of Health Ethical Committee (in Kwara, Kano, and Oyo States). In Tanzania, the study was approved by the Ifakara Health Institutional Review Board (IHI/IRB/NO. 44‐2021) and the National Institute of Medical Research (NIMR/HQ/R.8a/Vol.IX/3916). In addition, the team received permission letters to visit the selected sites from the President’s Office, Regional and Local Government.

All participants were informed about the study and signed a written consent before participation. Data were deidentified using unique ID numbers to maintain confidentiality. Participation was entirely voluntary, and no financial incentives were provided. Patients or the public were not involved in the design or conduct or reporting or dissemination plans of this research.

## Results

### Health Facility Assessment

We compare and summarize the health facility assessment findings from the 3 countries in Table 2 and present a heat map of these findings in Multimedia Appendix 1.

**Table 2.** Study findings from India, Nigeria, and Tanzania.

	Tanzania	Nigeria	India
Availability of policies and funds for essential health services			
Defined national SRH essential service package before COVID-19 pandemic	93.9 %	100 %	100 %
Identified core set of essential health services to be maintained during COVID-19 pandemic	63.6 %	83.3 %	100 %
Received additional government funding to assure essential health services	63.6 %	83.3 %	100 %
Received WHO^a^ guidelines on COVID-19 response and continuity of SRH^b^ essential health services	54.4 %	83.3 %	100 %
Maintenance of essential health services			
Outpatient department services	91 %	16.7 %	100 %
Inpatient services	57.6 %	16.7 %	54.5 %
Emergency health services	93.9 %	16.7 %	100 %
Prehospital emergency care services	93.9 %	0%	91 %
Community-based care	91 %	16.7 %	36.4 %
Mobile clinics	72.7 %	0%	Not applicable
Maintenance of essential SRH services as normal (no disruption)			
Contraception services	54.5 %	16.7 %	9.1 %^c^
Abortion services	66.7 %	0%	0%^d^
ANC^e^ services	66.7 %	33.3 %	63.6 %
Facility-based births	80 %	33.3 %	100 %
Routine immunization services	72.7 %	33.3 %	45.5 %
Sick child services/IMNCI^f^	75.8 %	33.3 %	81.8 %
Outbreak detection and control (for non-COVID diseases)	75.8 %	33.3 %	72.7 %
Inpatient critical care services	66.7 %	Not applicable	100 %
24 h emergency room	84 %	33.3 %	87.5 %
Availability of national FP^g^ and abortion guidelines/job aids and service referrals			
Availaility of national FP guidelines in facility	100 %	83.3 %	100 %
Availability of FP checklists or job aids in facility	100 %	100 %	100 %
Cilents referred for FP services to other health care facilities	48.5 %	0%	91.8 %
Availability of national abortion guidelines in facility	51.5 %	16.7 %	100 %
Availability of safe abortion checklists or job aids in facility	39.4 %	0%	100 %
Women who came to facility for postabortion care received FP counseling and services	57.6 %	50 %	69 %
Availability of contraceptive stocks (no stock out in the last 24 months)			
Combined oral contraceptive pill	48.5 %	16.7 %	90.9 %
Progestin-only contraceptive pill	46.9 %	33.3 %	Not applicable
Male condom	51.5 %	33.3 %	100 %
Female condom	43.8 %	16.7 %	Not applicable
Combined injectable contraceptives	63.3 %	16.7 %	Not applicable
Progestin-only injection	50 %	16.7 %	45.6 %
Centchroman	Not applicable	Not applicable	63.4 %
Cycle beads for standard days method	42.4 %	33.3 %	Not applicable
Vaginal ring	66.7 %	66.7 %	Not applicable
Subdermal implant	45.5 %	33.3 %	Not applicable
Copper IUD^h^	53.1 %	50 %	Not applicable
Levonorgestrel IUD	71.4 %	66.7 %	Not applicable
Diaphragm	78.9 %	33.3 %	Not applicable
Emergency contraceptive pill	41.9 %	50 %	100 %

a WHO: World Health Organization.

b SRH: sexual and reproductive health.

c Mainly due to the suspension of female sterilization services. Other contraceptive services were partially disrupted, leading to the upscaling of the community-based distribution of contraceptive commodities.

d Abortion services were available at only 2 of 11 facilities surveyed, and at both facilities, abortion services were partially disrupted.

e ANC: antenatal care.

f IMNCI: Integrated Management of Newborn and Childhood Illness

g FP: family planning.

h IUD: intrauterine device.

### Availability of Policies and Funds for Essential Health Services

Before the COVID-19 pandemic, all facilities in Nigeria and India had policies on the national SRH essential health services package, compared to 94% of facilities in Tanzania (Table 2). All health facilities in India, 83% in Nigeria, and 64% in Tanzania identified a core set of essential health services to be maintained during the pandemic. Additional government funds were allocated to all facilities in India, 64% of facilities in Tanzania, and 83% in Nigeria to ensure the continuation of essential health services. Approximately half of Tanzania’s facilities (54%) received the latest WHO guidelines on COVID-19 response and continuity of SRH services, compared to 100% in India and 83% in Nigeria. Overall, India demonstrated the highest preparedness and support for maintaining essential SRH services during the COVID-19 pandemic, with full policy coverage, guideline dissemination, and funding, whereas Tanzania lagged behind in all areas.

### Maintenance of Essential Health Services

During the COVID-19 pandemic, the outpatient department (OPD) services functioned normally in all Indian facilities, 91% in Tanzania, and only 17% in Nigeria (Table 2). The OPD services were suspended in one facility in Tanzania. Inpatient services continued to operate as usual in 55% of facilities in India, 58% in Tanzania, and 17% in Nigeria, with suspensions reported in 1 facility each in India and Nigeria. Emergency health services had minimal disruptions in India and Tanzania but major disruptions in Nigeria (only 17% functioned as normal). Community-based care continued normally in most facilities (91%) in Tanzania, 1 in Nigeria (17%), and 4 (37%) in India. Most mobile clinics functioned normally in 73% of Tanzania’s facilities, but none in Nigeria. In India, none of the included facilities offered mobile clinics. Thus, all essential health services were majorly disrupted in Nigeria, whereas India and Tanzania managed to maintain most services, with moderate disruptions in inpatient services.

### Maintenance of Essential SRH Services

Contraceptive services continued as normal in only 1 facility each in India (9.1%) and Nigeria (16.7%) and 18 facilities (54.5%) in Tanzania (Table 2). These services were partially disrupted by the pandemic in all 3 countries: 91% in India (mainly facility-based services such as sterilization services, which is the most common contraceptive method accepted overall in India); 83% in Nigeria; and 43% in Tanzania. One facility (3%) in Tanzania reported a complete disruption of contraceptive services, whereas no facility in India or Nigeria faced complete disruption of services. In fact, in India, outreach services were initiated through ASHAs for household distribution of condoms and OC pills. Abortion services were partially disrupted in both the facilities providing these services in India and Nigeria, and in 4 of the 15 (26.7%) facilities providing abortion services in Tanzania.

Antenatal care services were partially disrupted in 36% of facilities in India, 50% of facilities in Nigeria, and 33% of facilities in Tanzania. Facility-based births were not disrupted in India but experienced disruptions in 67% and 20% of facilities in Nigeria and Tanzania, respectively. Routine immunization services were partially disrupted in 46% of facilities in India, 67% in Nigeria, and 24% in Tanzania. One facility in India and Tanzania reported a complete disruption of immunization services. Inpatient critical care services were not disrupted in the Indian facilities, whereas they were partially disrupted in 33% of Tanzanian facilities. None of the included facilities in Nigeria offered inpatient critical care. In Nigeria, Tanzania, and India, emergency room services were partially disrupted in 67%, 16%, and 13% of the facilities.

Overall, Tanzania was the most successful among the 3 countries in maintaining essential SRH services during the pandemic, particularly contraceptive services, whereas India and Nigeria experienced partial disruptions, particularly in facility-based and abortion services. Nigeria was the most severely affected in maternal and child health services. Table 2 depicts the percentage of SRH services not disrupted during the pandemic.

### Availability of National FP and Abortion Guidelines/Job Aids and Service Referrals

National FP guidelines, checklists, job aids, and contraceptive stocks were available in all facilities in the 3 countries except for FP national guidelines in 1 Nigerian facility (Table 2). Referrals for FP services were made in 92% of Indian facilities (especially for female sterilization) and 49% of Tanzanian facilities, but none in Nigeria. National abortion guidelines and safe abortion checklists/job aids were available at all facilities in India, whereas in Nigeria, national abortion guidelines were available in one facility (17%), and none had safe abortion checklists/job aids. Tanzania had 52% of facilities with the national abortion guidelines, and 39% with the safe abortion checklists/job aids. FP counseling and services for postabortion care were offered in 50% and 58% of facilities in Nigeria and Tanzania, respectively. In India, 69% (majorly in urban areas) of women received sterilization and IUCD services after an abortion at a health facility. Overall, India had the most robust infrastructure supporting FP and abortion services, including widespread availability of national guidelines, job aids, and referral systems. Nigeria exhibited significant gaps, particularly in abortion guidelines, checklists, and the absence of FP referrals during the pandemic, whereas Tanzania had moderate availability of national guidelines and job aids but limited referral services.

### Availability of Contraceptive Stocks at Health Facilities

All 3 countries experienced contraceptive shortages during the pandemic (Table 2). Nigeria faced shortages of various contraceptives, including combined oral contraceptives (83%) and progestin-only pills (67%), injectables (both combined injectable contraceptives and progesterone only; 83%), male (67%) and female condoms (83%), and subdermal implants (67%). Approximately half of the facilities in Tanzania reported a shortage of both combined and progesterone-only pills, female condoms (56%), implants (55%), and emergency contraceptive pills (58%). India reported shortages of progestin-only injectables (55%) and centchroman (36%). Table 2 depicts the percentage of health facilities with no contraceptive stock out. Overall, Nigeria was the most severely impacted, with over two-thirds of facilities reporting shortages of multiple contraceptive methods. Tanzania experienced moderate contraceptive stockouts, whereas India reported fewer disruptions.

### Trends in Clients Seeking FP and Contraceptive Services

**Figure 1.**
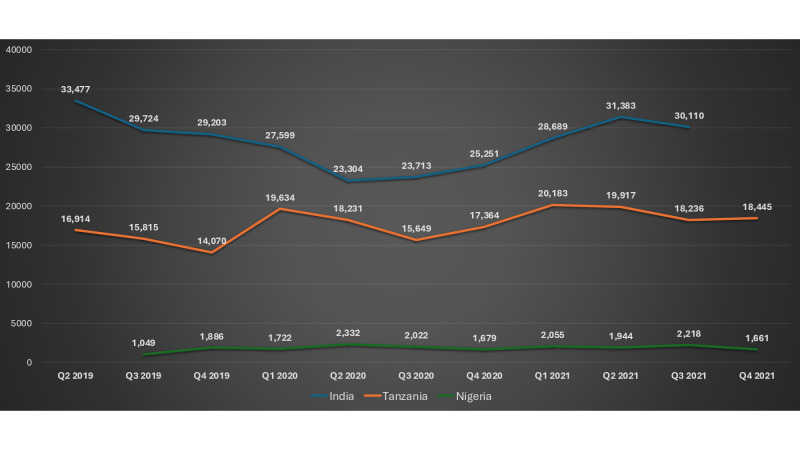
Trends in contraceptive uptake at the included health facilities in India, Tanzania, and Nigeria.

Data from health facilities were used to track trends of modern contraceptive uptake before and during the COVID-19 pandemic (Figure 1 and Table 3). India saw a 30% drop in clients visiting health facilities for contraceptive services during Q2 2020 (first wave of the pandemic; Table 3), then a recovery with a 35% increase in Q2 2021 compared to Q2 2020. Tanzania showed minimal change with a slight dip (1%) in Q3 2020, whereas Nigeria experienced an 11% decrease in clients in Q4 2020, with a recovery in Q1 2021, followed by a second dip in Q2 2021 (17%). Overall, India demonstrated a strong recovery following an initial sharp decline. Tanzania maintained consistent service use with minimal fluctuation, whereas Nigeria experienced more prolonged disruptions.

**Table 3.** Year-over-year percentage change in the contraceptive uptake at primary health facilities.

	Q1 2020 (%)	Q2 2020 (%)	Q3 2020 (%)	Q4 2020 (%)	Q1 2021 (%)	Q2 2021 (%)	Q3 2021 (%)	Q4 2021 (%)
India	^a^NA	−30.4	−20.2	−13.5	3.9	34.7	30	NA
Tanzania	20.1	7.8	−1	23.4	2.8	9.2	16.5	6.2
Nigeria	NA	NA	10.4	−11	19.3	−16.6	9.7	1

a Not applicable.

### Main Causes of Disruption or Change in Service Utilization

The causes of service disruption varied among the 3 countries (Table 4). In Nigeria, essential health services were mainly disrupted due to insufficient staffing at health facilities (83%), staff being assigned to COVID-19 services (83%), lack of public transportation (83%), and decreased number of clients at OPD (83%). India experienced disruptions due to a decrease in outpatient volume (54.5%) and lack of public transportation (45.5%). In Tanzania, service disruption was attributed to decreased outpatient visits (68.2%), insufficient staff (68.2%), and inadequate personal protective equipment (PPE) kits (63.6%). Overall, Nigeria experienced severe disruptions driven by workforce and access-driven constraints. India faced moderate challenges due to reduced outpatient volumes and mobility issues, whereas in Tanzania, a combination of decreased demand, staffing limitations, and insufficient PPE were predominant factors. Table 4 compares the major reasons for disruption or change in service usage during the pandemic.

**Table 4.** Reasons for disruption/ change in service utilization in India, Nigeria, and Tanzania.

Major reasons for the change in health service utilization	India (%)	Nigeria (%)	Tanzania (%)
Closure of OPD^a^ services	0	33.3	4.6
Closure of disease-specific consultation clinics	0	33.3	4.6
Decrease in OPD volume due to patients not presenting	54.5	83.3	68.2
Decrease in in-patient volume due to cancelation of elective care	18.2	50	22.7
Inpatient services are not available	18.2	0	0
Insufficient staff to provide services	36.4	83.3	68.2
Related clinical staff deployed to provide COVID-19 relief	36.4	83.3	50
Insufficient PPE available for healthcare providers	9.1	50	63.6
Stock out of essential medicines, medical diagnostics, or other health products	18.2	66.7	45.5
Changes in treatment policies for care-seeking behavior for fever symptoms	9.1	66.7	31.8
Government transport lockdowns hindering access to health facilities	45.5	83.3	18.2
Financial difficulties during outbreak/lockdown	18.2	66.7	27.3

a OPD: outpatient department.

### Approaches to Mitigate/Overcome the Disruptions to Essential Health Services

Countries adopted various measures to ensure the continuity of essential health services (Table 5). In India, Nigeria, and Tanzania, 91%, 83%, and 42% of facilities, respectively, implemented task shifting. Telemedicine services were provided by 82% of facilities in India, 50% in Nigeria, and 24% in Tanzania. When services were unavailable, patients were redirected to alternate health care facilities by all facilities in India, 67% of facilities in Nigeria, and 42% of facilities in Tanzania. In Nigeria, 83% of facilities informed the community about service disruptions and changes, whereas this was done by 42% and 18% of facilities in Tanzania and India. All facilities in India and 67% and 55% of facilities in Nigeria and Tanzania triaged clients to identify priority. In Tanzania, Nigeria, and India, user fees were removed in 6%, 17%, and 46% of facilities, respectively.

**Table 5.** Approaches used by countries to overcome disruption to essential health services during the pandemic.

Approach	India (%)	Nigeria (%)	Tanzania (%)
Telemedicine	81.8	50	24.2
Task shifting	90.9	83.3	42.4
Novel supply chain for medicines	54.5	33.3	3
Triaging to identify priorities	100	66.7	54.6
Redirection of patients to alternate health facilities	100	66.7	42.4
Community outreach to inform on service disruptions and changes	18.2	83.3	42.4
Government removal of user fees	45.5	16.7	6.1

## Discussion

### Principal Findings and Comparative Evidence

We determined the availability of contraceptive services in selected primary health facilities during the COVID-19 pandemic and compared the trends in contraceptive uptake before and during the pandemic in India, Nigeria, and Tanzania. Our study revealed that the COVID-19 pandemic resulted in partial disruptions in contraceptive services across the majority of health facilities in India and Nigeria and less than half of the facilities in Tanzania, particularly during the first wave of the pandemic. Notably, 1 facility in Tanzania even reported a complete disruption in contraceptive services. According to the WHO Pulse Survey, conducted in 2020 and 2021, 59% and 44% of countries, respectively, experienced partial disruptions, whereas 9% and 5% reported severe disruptions in FP and contraceptive services [19,20]. A study from South Asia showed a 3% to 31% decline in FP coverage during the period of stringent COVID-19 restrictions in 2020 [21]. Another study from sub-Saharan Africa showed a reduction in FP consultations by 10% to 25% between March and July 2020 in 4 of the 7 countries [22]. Our study noted a decline of 17% and 30% clients seeking contraceptive services in Nigeria and India during the initial wave of the pandemic in 2020, with a subsequent recovery in the number of clients. Literature suggests that despite initial declines during the pandemic, many countries were able to maintain or restore access to essential health services, including FP [13]. A multicountry cross-sectional study (Nepal, Uganda, Niger, and Malawi) reported that the pandemic reduced the number of long-acting reversible contraceptive users from 5% in Nepal to 22% in Malawi [23]. Contrary to the disruptions observed in India and Nigeria, our study showed a consistent increase in the usage of contraceptive services in Tanzania from 2019 to 2021. This upward trend could be attributed to Tanzania’s decision to refrain from imposing strict lockdown measures, allowing for unrestricted movement, which enabled them to maintain their FP clients. Similar results were reported from another study from Tanzania, which showed a slight dip in FP usage among existing users in 2020, followed by a rise in the number of new contraceptive users during 2020 and 2021 compared to the same period in 2019 [24]. All 3 countries experienced stock-outs of various contraceptives during the pandemic, affecting 36% to 83% of facilities. The United Nations Population Fund predicted in 2020 that most countries would experience stockouts of 1 or more modern contraceptive methods [8].

Our research identified several factors contributing to the disruption of contraceptive services during the COVID-19 pandemic. Main causes included a decrease in women visiting the facility, lack of public transportation, insufficient staff at health facilities, and staff being reassigned to COVID-19–related duties. These findings align with other studies that highlighted how the pandemic impacted women’s access to contraception due to supply chain disruptions, health providers being reassigned to COVID-related duties, and transportation issues caused by lockdowns [20,25-28]. According to the 2020 WHO Pulse survey, the main reasons for disruption in health services were patients not presenting to outpatient care (76%), transport lockdowns hindering access (48%), and financial difficulties during the pandemic (33%). On the supply side, the common causes were cancelation of elective care (66%), staff deployed to provide COVID-19 relief (49%), and insufficient PPE equipment for health providers (44%) [19]. A scoping review on barriers to accessing contraceptive services during the pandemic included fear of infection, lack of transport, cost, longer waiting times, reduced home visits by health workers, and limited availability of contraceptive supplies as the main barriers [2]. Findings from another study in Tanzania indicate factors for disruption in contraceptive services as changes in clinic schedules, fear of COVID-19 infection, and a shortage of contraceptives [24].

Countries implemented different approaches to overcome disruptions owing to the pandemic, such as redirecting clients to alternative health facilities, triaging to identify priorities, task shifting to lower-level health workers, using telemedicine, and conducting community outreach to inform clients about service disruptions. These strategies align with those reported in the WHO Pulse Survey, where triaging was the most common response (76%), followed by telemedicine (63%) and task shifting (57%) [19].

While our study offers valuable insights into how the pandemic affected contraceptive services and the strategies used by countries to address the crisis, it is essential to recognize that the study’s geographic coverage is limited, and the number of health care facilities examined was relatively small. These limitations could affect the generalizability of the study’s findings. Nonetheless, the study offers crucial insights into the challenges posed by the pandemic and the measures taken to mitigate them.

### Conclusions

Our study highlighted varying degrees of disruptions in essential health services and SRH services across the 3 countries. The most significant disruptions were observed in Nigeria, whereas Tanzania experienced comparatively fewer disruptions. Common factors contributing to service disruption in all 3 countries included reduced OPD visits and the lack of public transportation due to the lockdown. Mitigation strategies such as telemedicine, task shifting, triaging, community outreach, and redirection of patients proved effective in alleviating these disruptions. Moving forward, it is crucial for countries to implement and standardize these strategies to be better prepared for future pandemics.

## Supplementary material

10.2196/59874 Multimedia Appendix 1 Heatmap comparing study findings from India, Nigeria, and Tanzania

## Abbreviations

FP: family planning
PHC: primary health care
PPE: personal protective equipment
SRH: sexual and reproductive health
WHO: World Health Organization
