# Supplementary material for: Impact of the COVID-19 Pandemic on Contraceptive Services at Selected Primary Health Care Facilities in India, Nigeria, and Tanzania: Cross-Sectional Study
Source: Interact J Med Res. 2025 Dec 2;14:e59874. doi: 10.2196/59874 (PMC12683499; doi:10.2196/59874)
Supplement: Multimedia Appendix 1 [file ijmr-v14-e59874-s001.pdf]

	Tanzania	Nigeria	India
A. **Availability of policies and funds for essential health services**			
1. Defined national SRH essential service package before COVID-19 pandemic	91-100%	91-100%	91-100%
2. Identified core set of essential health services to be maintained during COVID-19 pandemic	61-70%	81-90%	91-100%
3. Received additional govt funding to assure essential health services	61-70%	81-90%	91-100%
4. Received WHO guidelines on COVID-19 response and continuity of SRH essential health services	51-60%	81-90%	91-100%
B. **Maintenance of essential health services**			
1. Out- patient department (OPD) services	91-100%	11-20%	91-100%
2. In-patient services	51-60%	11-20%	51-60%
3. Emergency health services	91-100%	11-20%	91-100%
4. Prehospital emergency care services	91-100%	0-10%	91-100%
5. Community based care	91-100%	11-20%	31-40%
6. Mobile clinics	71-80%	0-10%	not applicable
C. **Maintenance of essential SRH services as normal (no disruption)**			
1. Contraception services	51-60%	11-20%	0-10%*
2. Abortion services	61-70%	0-10%	0-10%#
3. ANC services	61-70%	31-40%	61-70%
4. Facility based births	71-80%	31-40%	91-100%
5. Routine immunization services	71-80%	31-40%	41-50%
6. Sick child services/IMNCI	71-80%	31-40%	81-90%
7. Outbreak detection & control (for non-COVID diseases)	71-80%	31-40%	71-80%
8. Inpatient critical care services	61-70%	not applicable	91-100%
9. 24-hour emergency room	81-90%	31-40%	81-90%
D. **Availability of National Family planning and abortion guidelines/job-aids and service referrals**			
1. Availability of National Family planning guidelines in facility	91-100%	81-90%	91-100%
2. Availability of FP checklists and/or job-aids in facility	91-100%	91-100%	91-100%
3. Clients referred for FP services to other health care facilities	41-50%	0-10%	91-100%
4. Availability of National abortion guidelines in facility	51-60%	11-20%	91-100%
5. Availability of safe abortion checklists and/or job aids in facility	31-40%	0-10%	91-100%
6. Women who came to facility for post-abortion care received FP counseling and services	51-60%	41-50%	61-70%
E. **Availability of Contraceptive stocks (no stock outs in last 24 months)**			
1 Combined oral contraceptive pill	41-50%	11-20%	91-100%
2 Progestin-only contraceptive pill	41-50%	31-40%	not applicable
3 Male condom	51-60%	31-40%	91-100%
4 Female condom	41-50%	11-20%	not applicable
5 Combined injectable contraceptives	61-70%	11-20%	not applicable
6 Progestin-only injection	41-50%	11-20%	41-50%
7 Centchroman	not applicable	not applicable	61-70%
8 Cycle beads for standard day method	41-50%	31-40%	not applicable
9 Vaginal ring	61-70%	61-70%	not applicable
10 Sub-dermal implant	41-50%	31-40%	not applicable
11 Copper IUD	51-60%	41-50%	not applicable
12 Levonorgestrel IUD	71-80%	61-70%	not applicable
13 Diaphragm	71-80%	31-40%	not applicable
14 Emergency contraceptive pill	41-50%	41-50%	91-100%

91-100% 81-90% 71-80% 61-70% 51-60% Below 50%

* Mainly due to the suspension of female sterilization services. Other contraceptive services were partially disrupted, leading to the upscaling of the community-based distribution of contraceptive commodities.
# Abortion services were available at only 2 out of 11 facilities surveyed, and at both facilities, abortion services were partially disrupted.
